# Changes in Fitness Parameters in Ridden Trained Showjumping Horses After Healing of Gastric Ulcers: Preliminary Results

**DOI:** 10.3390/vetsci13010009

**Published:** 2025-12-21

**Authors:** Sara Busechian, Alessandra Di Salvo, Simona Orvieto, Fabrizio Rueca, Chiara Villella, Gaia Sollevanti, Camillo Pieramati, Irma Nisi, Giorgia della Rocca

**Affiliations:** 1 Department of Experimental Medicine, Faculty of Medicine and Surgery, University of Rome Tor Vergata, Via Montpellier 1, 00133 Rome, Italy; sara.busechian@uniroma2.it; 2 Department of Veterinary Medicine, University of Perugia, Via San Costanzo 4, 06126 Perugia, Italy; fabrizio.rueca@unipg.it (F.R.); villechiara@gmail.com (C.V.); gaia.sollevanti98@gmail.com (G.S.); pieramati@alice.it (C.P.); irma.nisi@acmedrugs.eu (I.N.); giorgia.dellarocca@unipg.it (G.d.R.); 3 Independent Researcher, 06125 Perugia, Italy; simona.orvieto@gmail.com; 4 Acme Srl, Via Portella della Ginestra 9, 42025 Cavriago, Italy

**Keywords:** Equine Gastric Ulcer Syndrome, Equine Squamous Gastric Disease, Equine Glandular Gastric Disease, gastric ulcer healing, showjumping horses, stride length, speed, heart rate

## Abstract

Equine Gastric Ulcer Syndrome (EGUS) is a worldwide disease described in different categories of animals. A causal relationship between exercise and EGUS has been suggested, attributed to stress as well as physiological changes in the position of abdominal organs, especially during trot and gallop. EGUS can influence athletic fitness, as has been described in various papers on racehorses. The aim of this work was to determine if gastric ulcer healing is associated with changes in selected fitness parameters (i.e., speed, heart rate during exercise and recovery, stride length and frequency) in 21 trained showjumping horses affected by gastric ulcers. Four animals were excluded for incomplete healing or loss to follow-up. In the remaining seventeen subjects, all healed completely, and fitness parameters were evaluated before and after healing of EGUS during a ridden exercise. The animals were equipped with a fitness tracker validated for use in horses while they performed both flat work and a small obstacle course. Results show that speed and stride length increased after healing, with no concurrent modification in maximal heart rate recorded during exercise and the heart rate recorded during recovery. The increase in speed and stride length is probably related to the disappearance of abdominal discomfort, already described in racehorses after healing of gastric ulcers.

## Simple Summary

Equine Gastric Ulcer Syndrome is a worldwide disease with different prevalences described in various populations. Clinical signs are vague and non-specific, and many of the affected horses seem to be asymptomatic. The effect of the disease on fitness parameters has been studied in racehorses, but no information is available about showjumping animals. The aim of this study was to determine if healing of gastric ulcers changed fitness parameters in a population of 17 trained showjumping horses during a ridden exercise test. Omeprazole, associated with sucralfate in case of ulcers of the glandular mucosa, was used for treatment, and healing of the lesions was confirmed by gastroscopy. After healing, stride length and speed increased, indicating an improvement in fitness, potentially related to the disappearance of abdominal discomfort, as previously reported by other authors in racehorses.

## 1. Introduction

Equine Gastric Ulcer Syndrome (EGUS) is a common disease of the stomach of horses. The European College of Equine Internal Medicine (ECEIM) Consensus Statement, published in 2015, divided it into two different illnesses, based on the gastric mucosa involved: Equine Squamous Gastric Disease (ESGD) when the squamous mucosa is affected and Equine Glandular Gastric Disease (EGGD) when the glandular mucosa is involved [1,2,3,4]. The prevalence of EGUS is highest in racehorses, where more than 90% of animals are affected, especially during racing season and when in training for longer periods of time [1,2,3,4,5,6]. The disease has been diagnosed quite frequently also in other categories of horses, such as showjumping [7], pleasure [8,9,10], breeding [9,11], and western performance [12]. In a study performed on 83 jumpers, the prevalences of ESGD and EGGD were 39% and 70%, respectively, and according to the authors, the presence of the diseases was influenced by feeding and training intensity [7].

Clinical signs of EGUS are vague and nonspecific, such as ill-thrift, poor appetite, and recurrent colic; behavioral changes can also be associated with this syndrome, such as nervousness, aggression, teeth grinding, and girthiness. Many of the animals, though, show no clinical signs [1,2,3,4,9,13]. In addition, it has also been hypothesized that a decrease in the horse’s performance may be a clinical sign of EGUS [4].

The effect of EGUS on horse performance is currently not completely elucidated, but several reports indicate that the presence of gastric ulcers can potentially be correlated with racing below expectations and decreased performance in racehorses [2,5,14,15,16,17]. Recently, the severity of ESGD was associated with a decline in racing results, evaluated as the number of placings [15]. EGUS, especially ESGD, was associated with worse fitness parameters in both Thoroughbreds and Standardbreds exercising on a treadmill [15,17,18]. In particular, following a training program that involved progressive increases in exercise, the mass-specific maximal O2 consumption was significantly higher in horses without ulcers compared to horses with EGUS, and this last category was not able to improve their aerobic capacities as much as the healthy ones after the training program [18]. Similarly, in Standardbreds submitted for evaluation of poor performance and running on a treadmill, severe forms of ESGD have been associated with lower speed, measured both at a heart rate of 200 bpm and at a plasma lactate concentration of 4 mmol/L, suggesting that gastric ulcers may compromise these fitness parameters [17].

Conversely, a case report showed that the athletic performance of four Thoroughbred racehorses affected by EGUS improved following healing [14]. Furthermore, according to the authors’ experience, after healing from EGUS, horses appeared more relaxed and had a better attitude towards exercise, with some of the riders reporting empirically longer strides in racehorses.

Exercise testing is a field of research in equine sports medicine, and various instruments have been validated for use in horses so that physiological parameters can be recorded while the horse is exercising. Both in research and when diagnosing poor performance, it is important to adapt the type and intensity of the exercise test to the competition the animals will be performing: this will guarantee that the results can be correlated with the normal exercise performed by the horses [19,20]. Various devices have been studied and marketed to record different parameters in exercising horses: almost all of them register speed, position, and heart rate, some are also able to evaluate other parameters or use artificial intelligence and machine learning algorithms to determine other factors, such as stride length or symmetry of the gait, but also to record exercising ECG that help to determine the presence of cardiac arrhythmias, only present during exercise [19,20,21]

A prevalence of gastric ulcers in showjumpers between 60 and 80% has been reported, with the higher percentage found in elite horses during the sport season [7,22]. To the authors’ knowledge, no information is available about the effect of EGUS on fitness parameters in this category of sport animals. The aim of this study was therefore to determine the possible effect of healing of gastric ulcers, following treatment, on fitness parameters in trained showjumping horses during exercise in the field.

## 2. Materials and Methods

The present study was approved by the Bioethical Committee of the University of Perugia (on 26 July 2022, with the protocol number 21/2022). All owners of enrolled horses gave their written informed consent to participate in the study.

Trained adult showjumping horses referred by veterinarians, owners, and riders on the basis of the presence of risk factors or of clinical signs compatible with EGUS [3,4,9,23] were evaluated for inclusion. Reasons for exclusion were the presence of systemic diseases and lameness in the two months before enrollment and treatment with any kind of medication in the last month. Furthermore, to reduce biases related to training and rider, horses were excluded if they were in training for less than two years or if a change of rider was reported in the six months before the inclusion. During the treatment period, horses were excluded if any clinical sign of systemic disease or lameness appeared, and if the training protocol was changed in any way (increased or decreased intensity, change of rider or training facility). Riders had to have at least two years of experience in showjumping, and the horse–rider combination must have performed at least 1 m jumping courses for at least the last six months before inclusion.

After a clinical examination, a gastroscopy was performed according to the literature, using a 3 m scope (60130PKS, Karl Storz, Tuttlingen, Germany) and a portable processor (Tele Pack Vet X Led, Karl Storz, Tuttlingen, Germany). The animals were sedated with xylazine (0.6–1 mg/kg IV) and restrained with a twitch. The endoscope was introduced through the ventral nasal meatus into the pharynx and the esophagus to reach the stomach. Here, all the regions were visualized, dilating the organ with air and washing the mucosa with distilled water when necessary. The video of the endoscopy was recorded on a USB stick for evaluation and storage. Lesions of the squamous mucosa were graded according to the literature using a 5-point scale (grades 0 to 4) [1], while those of the glandular mucosa, due to the lack of a validated grading system, were considered positive for EGGD in presence of any alteration of the glandular mucosa (e.g., hyperemia, erosions or ulcers, raised or flattened lesions) [1,2].

After the gastroscopy, horses presenting lesions of the squamous mucosa of at least grade 3 were subjected to a ridden exercise test while wearing a fitness tracker device validated for use in equines (Equimetre, Arioneo, Le Bouscat, France) [24,25]. Before the start of the exercise session, clinical and lameness examinations were carried out to exclude the presence of overt clinical diseases that developed in the timeframe between gastroscopy and the first ridden exercise. The training session consisted of 15 min of flat work at all gaits, a small course of 4 obstacles whose height was determined by the level of experience and training of both rider and horse, and 5 min of recovery performed at trot and walk. The applied exercise protocol was previously described to evaluate changes in the ridden horse pain ethogram in horses with gastric ulcers subjected to a diet modification [26,27]. The rider was asked to perform at least five minutes at each gait and to push the horse to the highest speed possible for the entire length of the riding arena for at least one lap. In the days following the training, the ECG signal recorded by the device was examined offline to exclude the presence of severe cardiac arrhythmias developing during exercise (e.g., atrial fibrillation). If horses presented lameness or cardiac arrhythmias during exercise, they were excluded from the study.

The enrolled animals were treated with oral omeprazole (4 mg/kg once daily) for 30 days, associated with sucralfate (12 mg/kg twice daily) if EGGD was present. The owners were instructed to administer omeprazole on an empty stomach, at least 30 min before the morning meal, and the sucralfate. At the end of the treatment, clinical and gastroscopic examinations were performed again as previously described.

At the follow-up gastroscopy, horses were considered healed if ESGD was graded 0 or 1, improved if graded 2, and not responding if the ESGD grade was not changed or increased. Only healed animals were subjected to a second clinical and lameness examination and to a new ridden exercise test as previously described, to avoid bias related to the presence of gastric lesions that could influence the horses’ welfare.

Both exercise tests (before and after therapy) were conducted by the same rider, using the same riding arena and the same tack, to exclude biases related to environment and rider. The sequence of the gaits and the height and type of jumps were also maintained as similar as possible between the two exercise tests to avoid biases related to increased or decreased exercise intensity.

At the end of each training session, the fitness tracker device was synchronized with the smartphone app provided by the manufacturer, and all data were stored online. Fitness parameters of each horse were calculated automatically by the system and downloaded to an Excel sheet. The fitness tracker recorded heart rates continuously during the test, together with the speed and GPS position of the animals. Using artificial intelligence, the device calculates different parameters, such as gaits, stride length and frequency, together with heart rates during exercise and recovery.

Due to the lack of information available in the literature on the effect of gastric ulcers and their treatment on fitness parameters in showjumping horses, the power of the study was determined “a posteriori,” using the means and standard deviations of the fitness parameters recorded in the two groups (before and after treatment). The power of the study was above 70% for all parameters evaluated. The analysis was performed using G*Power 3 [28]. Kolmogorov–Smirnov test was used to investigate the normal distribution of the variables, and based on the results, differences in the fitness parameters recorded before and after treatment were evaluated using the most appropriate test. A t-test was applied for normally distributed parameters and a Wilcoxon signed-rank test for paired samples for those not normally distributed. Statistical significance was set at *p* < 0.05, and the analysis was performed using RStudio (version 2025.05.1) [29].

## 3. Results

**Figure 1:**
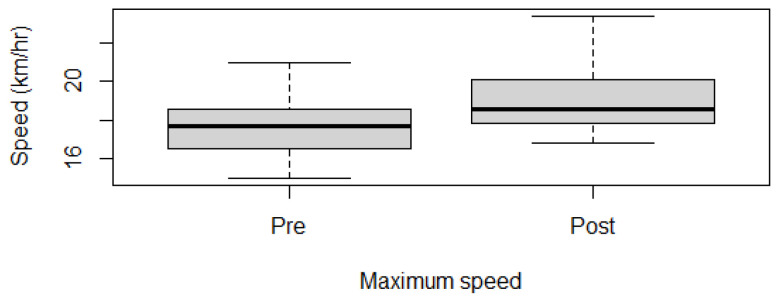
Maximal speed recorded in this population of 17 healed showjumping horses. Pre: before treatment, Post: after treatment.

Twenty-one horses were included in the study. Animals were of different breeds and sexes and aged between 7 and 22 years (median 11, interquartile range: 9–13). Table 1 and Figure 1 show the distribution of the animals according to their signalment. Table 1 also reports the results of the ESGD scores and the presence/absence of EGGD in enrolled horses before and after treatment with omeprazole.

At inclusion, 8/21 (38%) horses presented ESGD grade 3 and 13/21 (62%) grade 4; EGGD was detected, as areas of hyperemia of different extensions, in 8/21 (38%) animals. After treatment, all horses were negative for EGGD, while, according to ESGD scores, 17/21 (81%) were healed, 3/21 (14%) were improved, and 1/21 (5%) did not respond to the therapy. The owners of the improved (but not completely healed) animals declined further treatment, while the pony that did not respond, despite continuing the treatment, was lost at follow-up. Thus, these four subjects were not included in the statistical analysis and were excluded from the study.

**Figure 2:**
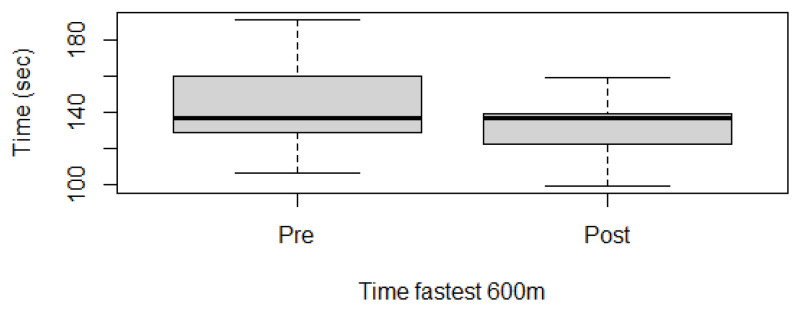
Time necessary to run the fastest 600 m in this population of 17 healed showjumping horses. Pre: before treatment, Post: after treatment.

**Figure 3:**
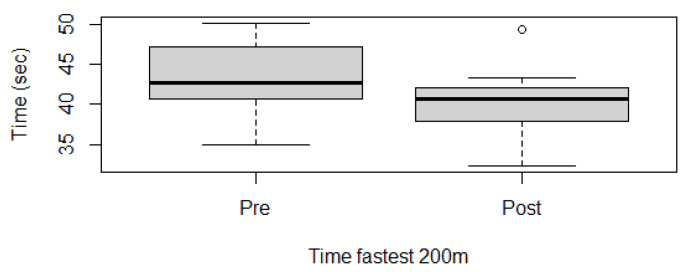
Time necessary to run the fastest 200 m in this population of 17 healed showjumping horses. Pre: before treatment, Post: after treatment.

**Figure 4:**
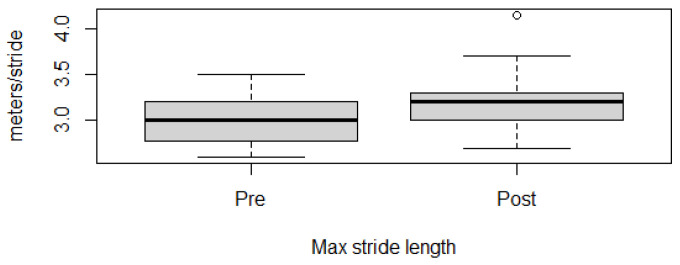
Maximal stride length recorded in this population of 17 healed showjumping horses. Pre: before treatment, Post: after treatment.

**Figure 5:**
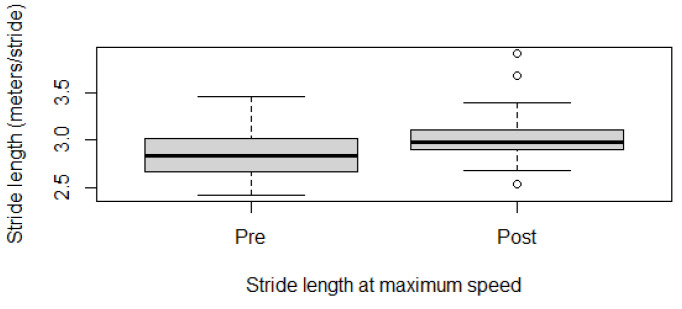
Stride length recorded at maximal speed in this population of 17 healed showjumping horses. Pre: before treatment, Post: after treatment.

**Figure 6:**
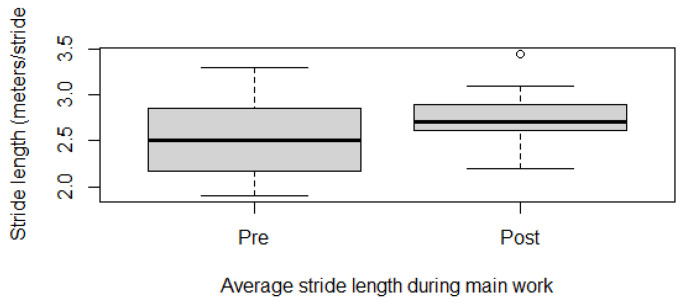
Average stride length recorded during the main work in this population of 17 healed showjumping horses. Pre: before treatment, Post: after treatment.

**Figure 7:**
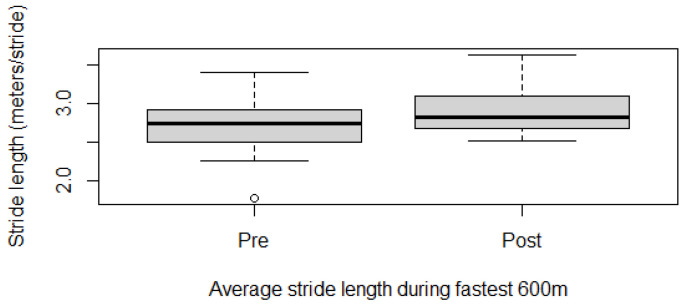
Average stride length recorded during the best 600 m in this population of 17 healed showjumping horses. Pre: before treatment, Post: after treatment.

**Figure 8:**
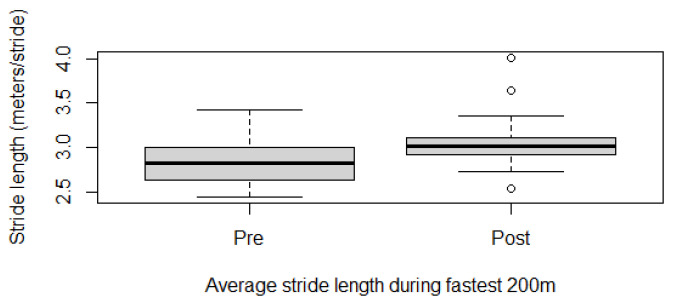
Average stride length recorded during the best 200 m in this population of 17 healed showjumping horses. Pre: before treatment, Post: after treatment.

Fitness parameters recorded by Equimetre at inclusion and after 30 days of treatment, as well as results of the statistical analysis performed on data recorded before and after treatment, are shown in Supplementary Materials Table S1. The distribution of the data was not normal for all the evaluated parameters, so the Wilcoxon signed-rank test for paired samples was used to compare the different parameters at the two time points. Statistically significant differences between the tests performed before and after healing were found for the following parameters: max speed reached during exercise (*p* = 0.02, Figure 1), time necessary to run the fastest 600 m (*p* = 0.02, Figure 2) and 200 m (*p* = 0.001, Figure 3), maximal stride length recorded (*p* = 0.01, Figure 4), stride length at maximal speed (*p* = 0.02, Figure 5), average stride length during the main work (*p* = 0.03, Figure 6), and average stride length during the fastest 600 m (*p* = 0.03, Figure 7) and 200 m (*p* = 0.004, Figure 8). No changes were seen for the other recorded parameters (working duration and distance, heart rate recorded during exercise, both maximal, at the end, and during recovery, heart rate, speed, and regularity of the first trot and first canter). 

## 4. Discussion

Equine Squamous Gastric Disease (ESGD) and Equine Glandular Gastric Disease (EGGD) are defined as the presence of lesions in the squamous and glandular mucosae of the stomach of equids [1,2,3]. They are present worldwide, with various prevalence in different categories of horses, the highest described in racehorses in active training [1,2,3,4,6,30]. The clinical significance of EGUS is not completely elucidated, because most of the animals do not show overt signs of the disease, or, if they do, these can be vague and nonspecific (e.g., recurrent colic responsive to medical management, weight loss, etc.) [3,4,9]. The relationship between gastric ulcers and poor performance has been investigated in racehorses, where the presence of the disease has been associated with exercise intolerance and racing below expectations in some studies [5,15,17]. The reasons for this association have only been postulated in some studies concerning racehorses, where the authors have correlated the reduced performance to the presence of abdominal discomfort related to the disease. This can cause a decrease in the stride length but also reduced diaphragmatic excursion and, consequently, altered gas exchanges at the alveolar level, decreased aerobic performance, and increased use of anaerobic metabolism and lactate production [13,15,16,18]. To the authors’ knowledge, no information about the effect of gastric ulcers on performance in showjumping horses is available so far. The aim of this study was to evaluate the presence of possible changes in fitness parameters after healing of gastric ulcers in jumpers.

Performing a jumping course requires a combination of aerobic and anaerobic exercise: the former is used during the cantering between obstacles and the latter during the jump itself, especially in poorly trained animals, when the heart rate increases above that of the rest of the effort. Heart rate during a jumping course was recorded between 180 and 206 bpm, with higher values found at the end and when jumping a spread obstacle compared to an upright obstacle. Recovery starts immediately after the last jump. Lactate concentrations have been reported between 3 and 8 mmol/L after jumping. Based on the metabolic findings, it has been suggested that, to evaluate the performance of these horses, it is important to include in the test also a few jumps, especially when considering subclinical diseases [22,31,32].

Fitness evaluation of exercising horses has been a field of research for a long time. Both treadmill and field tests have been proposed, and currently, the latter are made more affordable and easier to perform using portable instrumentation that can be applied in racetracks and riding arenas [17,18,19]. These devices allow to register different parameters at the same time, such as speed, heart rate, stride parameters, and symmetry. Some of them record ECG [17,18,19,20,21], which can be useful to detect arrhythmias but also to evaluate the heart rate variability (HRV). The information gathered can be used to identify subclinical diseases but also to monitor and guide training regimens to achieve the best performance from each horse [18,22,23,24].

In our study, field tests were performed. Even if some authors demonstrated that the physiological demands of a jumping course are similar to those of an exercise on a track, to better represent the real condition of showjumping horse work, a jumping course was included in the test [22,25]. During the month of treatment, the training regimen of the horses did not change and was consistent with the one performed by horse and rider during the six months before inclusion.

The population of animals enrolled in this study was reflective of the current breeds and sexes employed in showjumping: warmblood geldings are usually used, especially in lower-level competitions [22]. The age range in this study was wider than what is typically found in sport horses, but a recent survey administered to owners in the US showed that older animals are still used for competition in various disciplines [33]. In our population, two of the oldest horses (20 and 22 years), even if they were not competing, were still trained two or three times a week to maintain their fitness level. The 20-year-old pony, similarly, was still shown on low-level obstacle courses less than once a month. These old horses responded to all the inclusion criteria indicated in the study, and the owners did not report any signs of discomfort during training.

Healing of gastric ulcers improved some fitness parameters in this population of showjumping horses. The speed was significantly increased after treatment. The increase in the maximal speed reached during the exercise is correlated with the significant decrease in the time spent running the fastest 600 m and 200 m. Speed has been previously correlated with fitness level in various studies [17,19,34,35,36]. In particular, it is usually associated with the evaluation of lactic acid concentrations or heart rate to determine the workload and also the adaptation to training in various sport horses [19,34]. V170 and V200, the speed at which the athlete reaches 170 and 200 bpm, respectively, are considered reliable indicators of fitness in sports medicine. Evaluating these parameters in regular tests can help identify subclinical problems but also check the adaptation to training. The higher the two values, the better the response of the athlete to the proposed exercise [19,34]. In the population evaluated in the present study, these two parameters have not been calculated; nevertheless, the not significant difference in the maximal heart rate recorded before and after healing, associated with an increase in maximal speed, indicates that the improvement in this fitness parameter could be associated with the healing of the gastric ulcers. This hypothesis would also be in line with the literature, where EGUS, especially ESGD, was associated with a lower V200 value in poorly performing Standardbreds on a treadmill [15]. Nevertheless, it is important to underline that this last study did not evaluate the horses after treatment, and some of the animals also presented mild asthma, which could also have an impact on fitness parameters [37,38].

The increase in speed observed in the present study is almost certainly due to an increase in the stride length and not to a change in stride frequency. In fact, in this study, statistically significant differences have been found for stride length recorded in different moments during exercise: the maximal stride length, the stride length at maximal speed, and the average stride length during the main work and during the fastest 600 and 200 m. These findings are in concordance with the observations in Thoroughbreds exercising on a treadmill, where experimental induction of gastric ulcers was associated with a lower increase in stride length with training, compared to healthy controls. According to the authors, this difference was related to abdominal discomfort associated with the presence of the disease [18]. An increase in stride length following EGUS healing has also been reported to the authors of the present study by some owners of racing Thoroughbreds. Abdominal discomfort in horses with gastric ulcers has been associated with the presence of girthiness and other behavioral abnormalities while saddling; these behavioral aspects, easily identifiable by the owners, could be used as a warning to investigate the animal’s health, especially when associated with reduced performance [13].

Decrease in stride length has been associated with fatigue in different disciplines [39,40,41]; however, in a recent paper, the length of strides was not associated with race outcome in a group of Thoroughbreds in training [42]. A different paper, though, showed that decreases in stride length and speed over multiple starts were associated with increased risk of musculoskeletal injury [35]. Such information is not available for showjumpers, and further studies are needed to determine if the change in stride length could be associated with the delayed onset of fatigue, also in this population of horses. Furthermore, in showjumping, stride length is directly related to the ability of the horse to clear the obstacles [22]: an inability to increase the stride length, secondary to abdominal discomfort, could potentially lead to decreased performance in this population. Further studies are also needed to confirm if the changes in stride length are related to an improvement in the results of the shows, especially in high-level competition animals.

As it has been demonstrated that omeprazole therapy does not have an effect on fitness parameters in healthy, ulcer-free Standardbreds exercised on a treadmill [43], the improvement in fitness parameters observed in this study is related with reasonable certainty to the healing of ESGD and EGGD. Furthermore, the ridden test was performed at least 48 h after the end of the treatment, when pharmacokinetic studies have demonstrated that the drug is no longer detectable in blood, even after 29 days of treatment [44]. At the same time, as horses were trained for showjumping and maintained at the same level of exercise throughout the whole study period, an effect of training on these parameters, if present, can be considered minimal.

The study has some limitations. First, it is not possible to exclude that, despite having been asked to perform at the top of their horses’ ability in both tests (before and after the therapy), the riders could have been more willing to ask for higher speeds during the second test (after healing) because they were aware that the ulcers were not present anymore. Nevertheless, they could not control the stride length and the heart rate of their animals. Furthermore, the riders did not have access in real time to the parameters recorded by the Equimetre and could not have been influenced by these results. In future studies, the ridden test could be performed before the gastroscopy, or riders could be blinded to the results of the examination to exclude this potential bias.

Another limitation could be the inclusion of older subjects that were not showing anymore; however, as previously stated, the horses were still in training for showjumping and regularly ridden by their owners. No specific tests were carried out to determine the presence of subclinical diseases, but a complete history was collected, and clinical and lameness examinations were performed before the start of each ridden exercise to exclude overt respiratory, cardiovascular, and orthopedic diseases that could have influenced fitness parameters.

Finally, this study lacks a control group, as, for ethical reasons, it is not possible to voluntarily withhold treatment in horses with grade 3 and 4 ulcers. Therefore, it was only possible to use each horse as its own control, maintaining stable throughout the study rider, tack, riding arena, and training regimen to avoid any outside influence on fitness parameters when comparing the exercise before and after treatment.

## 5. Conclusions

In this population of trained showjumping subjects, the healing of EGUS was associated with improvement in some fitness parameters, such as maximum speed reached due to an increase in the stride length. Maximal heart rate did not change between before and after treatment. These data indicate that healing of gastric ulcers could influence selected fitness parameters (stride length and speed) during exercise, probably related to the disappearance of abdominal discomfort associated with the disease, but further studies are needed to confirm these findings, due to the preliminary nature of this research.

## Figures and Tables

**Table 1:** Signalment and results of the gastroscopies of the 21 horses.

Breed	Age	Sex	ESGD_t1	ESGD_t2	EGGD_t1	EGGD_t2
Italian Saddlebred	13	male	3	0	neg	neg
German Saddlebred	8	gelding	4	0	pos	neg
German Saddlebred	14	gelding	3	0	pos	neg
Pony	17	gelding	4	0	pos	neg
French Saddlebred	7	gelding	4	0	neg	neg
Belgian Saddlebred	7	gelding	4	0	pos	neg
Belgian Saddlebred	9	gelding	3	0	neg	neg
Warmblood	12	gelding	4	0	neg	neg
Dutch warmblood	11	gelding	3	0	neg	neg
Irish warmblood	11	gelding	3	0	neg	neg
Pony	20	female	3	0	neg	neg
French Saddlebred	12	gelding	3	0	neg	neg
Italian Saddlebred	10	gelding	4	2	neg	neg
German Saddlebred	9	gelding	4	2	pos	neg
German Saddlebred	11	gelding	4	0	pos	neg
Dutch warmblood	17	gelding	4	0	pos	neg
Italian Saddlebred	8	female	4	0	neg	neg
French Saddlebred	11	male	3	0	neg	neg
Belgian Saddlebred	11	gelding	4	2	neg	neg
Dutch warmblood	22	female	4	0	pos	neg
Pony	12	gelding	4	4	neg	neg

t1: gastroscopy at inclusion; t2: gastroscopy after 30 days of treatment with omeprazole. ESGD: Equine Squamous Gastric Disease, EGGD: Equine Glandular Gastric Disease, pos: glandular mucosa showing any alteration, neg: normal glandular mucosa appearance.

## Data Availability

The raw data supporting the conclusions of this article will be made available by the authors on request.

## Supplementary Materials

The following supporting information can be downloaded at https://www.mdpi.com/article/10.3390/vetsci13010009/s1, Table S1: Median and interquartile range (IQR) of parameters recorded by Equimetre at inclusion time (t1) and after 30 days of treatment with omeprazole (t2). In the last column, the *p*-value following statistical analysis is reported.
