# Supplementary material for: Changes in Fitness Parameters in Ridden Trained Showjumping Horses After Healing of Gastric Ulcers: Preliminary Results
Source: Vet Sci. 2025 Dec 21;13(1):9. doi: 10.3390/vetsci13010009 (PMC12846468; doi:10.3390/vetsci13010009)
Supplement: Supplementary file 1 [file vetsci-13-00009-s001.zip › vetsci-4043300-supplementary.pdf]

Table S1. Median and interquartile range (IQR) of parameters recorded by Equimetre at inclusion time (t1) and after 30 days of treatment with omeprazole (t2). In the last column, p value following statistical analysis is reported.

Parameter Equimetre	t1 median (IQR)	t2 median (IQR)	p
Working duration (sec)	167.00 (159.00-247.00)	182 (156-284)	0.75
Working distance (m)	686.00 (597.00-838.00)	757 (570-890)	0.54
Maximal speed reached during the exercise (km/hr)	17.7 (16.5-18.6)	18.3 (17.1-19.5)	**0.02**
Time to run best 600 meters (sec)	145 (132-165)	137 (130-138)	**0.02**
Time to run best 200 meters (sec)	43 (41-49)	41 (39-42)	**0.001**
Maximal heart rate reached during training (bpm)	155 (131-162)	148 (137-164)	0.39
Heart rate at the end (bpm)	48 (46-54)	52 (48-59)	0.19
Heart rate at the end as percentage of maximal heart rate (%)	22 (21-25)	24 (22-27)	0.2
Heart rate after 1 minute of recovery (bpm)	101 (81-115)	103 (99-110)	0.45
Heart rate after 1:30 minutes of recovery (bpm)	97 (76-115)	96 (74-103)	0.93
Heart rate after 2 minutes of recovery (bpm)	98 (76-116)	92 (73-102)	0.56
Heart rate after 3 minutes of recovery (bpm)	80 (70-98)*	91 (69-111)	0.54
Heart rate after 4 minutes of recovery (bpm)	81 (69-96)*	74 (65-105)	0.78
Heart rate after 5 minutes of recovery (bpm)	83 (64-94)*	83 (65-113)	0.71
Maximal stride frequency recorded (stride/s)	1.74 (1.72-1.82)	1.74 (1.70-1.84)	0.75
Maximal stride length recorded (m)	3.00 (2.75-3.20)	3.20 (3.00-3.30)	**0.01**
Stride frequency at maximal speed (stride/sec)	1.72 (1.68-1.80)	1.71 (1.68-1.77)	0.59
Stride length at maximal speed (m)	2.83 (2.52-3.01)	2.96 (2.88-3.09)	**0.02**
Average stride length during the main work (m)	2.31 (2.10-2.80)	2.70 (2.50-2.90)	**0.03**
Average stride length during the fastest 600 meters (m)	2.73 (2.42-2.86)	2.81 (2.65-3.10)	**0.03**
Average stride length during the fastest 200 meters (m)	2.83 (2.54-2.95)	3.02 (2.92-3.09)	**0.004**
Mean heart rate during the first trot (bpm)	102 (95-108) §	96 (91-101)*	0.32
Mean speed reached during the first trot (km/hr)	11.4 (10.7-11.7) §	10.8 (9.2-11.8)*	0.54
Mean heart rate during the first canter (bpm)	129 (118-137) §	129 (120-139)*	0.35
Mean speed reached during the first canter (km/hr)	15.6 (14.1-16.4) §	15.6 (14.5-16.5)*	0.53
Mean regularity of the strides during the first canter (%)	97 (96-97.5) §	97 (97-98)*	0.72

* data available for 16 horses; § data measured on 15 horses.
